# Supplementary material for: Peri-Urban Community Attitudes towards Codling Moth Trapping and Suppression Using the Sterile Insect Technique in New Zealand
Source: Insects. 2019 Oct 9;10(10):335. doi: 10.3390/insects10100335 (PMC6835363; doi:10.3390/insects10100335)
Supplement: Supplementary file 1 [file insects-10-00335-s001.pdf]

Peri-Urban Community Attitudes towards Codling Moth Trapping and Suppression Using the Sterile Insect Technique in New Zealand

Georgia Paterson [1], George LW Perry [2], James TS Walker [3] and David Maxwell Suckling [4,*]

S1. Questionnaire presented to participants.

Survey on attitudes to aspects of the sterile insect technique:
This is a University of Auckland MSc study with Plant and Food Research entomologists, working on novel ways of controlling insects without pesticides. Hawke's Bay growers already use sex pheromones against the moth pests in apple orchards and export residue-free fruit, which is great because it supports the economy without insecticides. Now, we are releasing sterile codling moths to overflood the remaining wild populations. We have a few questions to better understand the community's view on what trying to make codling moth go extinct might look like. This information will be managed according to University of Auckland policy and not be available beyond the researchers. Submission of the questionnaire counts as consent to the participation. Due to the anonymity of the information, data cannot be withdrawn once the questionnaire has been submitted.
Here are my 16 questions:
1. Gender M/F
2. Age decade 11-20 (1), 21-30 (2), 31-40 (3), 41-50 (4), 51-60 (5), 61+ (6), Prefer not to answer (0)
3. Does a member of your family work in horticulture? Yes (1) No (0)
4. Are you concerned about invasive pest mammalian species? 1 (not) - 5 (very open)
5. Are you concerned about invasive pest insect species?
6. Do you have an apple tree at this location? Yes (1) No (0)
7. Do you have a walnut tree at this location? Yes (1) No (0)
8. Do you harvest the apples? Yes (1) No (0)
9. Do you have codling moth, the "worm in the apple"? Yes (1) No (0)
10. We are proposing the release of harmless sterile moths against the local pest codling moths, using a small, lightweight unmanned aerial vehicle, with a wing span of 2.5 m. It is quiet and flies high overhead and can deliver 100 ha pest free without any insecticides in 10 minutes. Are you open to the idea? 1 (not) - 5 (very open)
11. Would you be open to this technology being used during an emergency fruit fly or similar insect pest response? 1 (not) - 5 (very open)
12. UAVs can be based on octocopters or fixed wing. Are you open to the idea of octocopters? 1 (not) - 5 (very open)
13. Do have any reservations about this proposed method? Yes (1) No (0)
And if so, what are they?
14. Would you be open to a pheromone trap being set up on your apple tree? Yes (1) No (0)
15. Would you consider the removal of your infected tree in exchange for a gift or incentive? Yes (1) No (0)
16. Did you have any further questions or comments? Yes (1) No (0)

S2. Participant Information Sheet
Project title: Can codling moth (*Cydia pomonella*) be eradicated from peri-urban Hawke's Bay?
Name of Supervisor: Professor George Perry
Name of Student Researcher: Georgia Paterson
Researcher introduction and project description
This is a University of Auckland MSc study with Plant and Food Research entomologists, working on novel ways of controlling insects without pesticides. Student researcher Georgia Paterson will primarily be conducting this research.
Hawke's Bay growers already use sex pheromones against the moth pests in apple orchards and export residue-free fruit, which is great because it supports the economy without insecticides. Now, we are releasing sterile codling moths to overflood the remaining wild populations. We have a few questions to better understand the community's view on the efforts and methods involved in this process. This information will be managed according to University of Auckland policy.
Project invitation and procedures

You are invited as a participant to be involved in the research. You have been selected to participate in this questionnaire, as the proposed research is to take place in your area. This key aim of this study is to investigate whether a reduction in the peri-urban populations of codling moth is likely to be possible through pheromone trapping and future sterile releases. The success of an area-wide eradication programme relies upon a holistic approach, which emphasises the need for community participation. The questionnaire will give an indication of the communities' response to the research. Anonymity will be guaranteed and all details given in the questionnaire will remain anonymous. Participation is completely voluntary. Participant's names are not being collected and will therefore not be attached to the questionnaire to ensure anonymity.

The questionnaire is made up of ten questions, most of which will be answered by rating your response from 1 to 5. This will take approximately ten minutes in total.

Data storage/retention/destruction/future use

All data from this questionnaire will be stored securely, and identifying materials (including key words or codenames) should be stored separately from coded data. This will be stored electronically for six years then subsequently destroyed.

Anonymity and Confidentiality

Anonymity within the questionnaire is guaranteed. This will be done through assigning each participant response a code. This data will be available to the researchers involved in the study, however all details will remain confidential.

If the information you provide is reported or published, this will be done in such a way that its source cannot be identified. Withdrawal of data will not be possible as it will not be known what data you have directly provided.

Contact Details and Approval Wording

If more information about the study is required, you are invited to contact the HOD, supervisor and/or researcher

Professor Paul Kench (p.kench(@)auckland.ac.nz, +64 9 373 7599 ext 88440)

Professor George Perry (g.perry(@)auckland.ac.nz)

Georgia Paterson (gpat568 (@) aucklanduni.ac.nz)

For any concerns regarding ethical issues you may contact the Chair, the University of Auckland Human Participants Ethics Committee, at

The University of Auckland Research Office,

Private Bag 92019,

Auckland 1142.

Telephone 09 373-7599 ext. 83711.

Email: ro-ethics(@)auckland.ac.nz
